# Vertically aligned nanostructured gold microtube assisted by polymer template with combination of wet phase inversion and Cu grid mask

**DOI:** 10.1038/s41598-020-73506-1

**Published:** 2020-10-02

**Authors:** Soohyun Kim, Keon-Soo Jang

**Affiliations:** 1 LG Chem, Magokjungang 10-ro, Gangseo-gu, Seoul, 07796 Republic of Korea; 2 Department of Polymer Engineering, School of Chemical and Materials Engineering, The University of Suwon, Hwaseong, Gyeonggi-do 18323 Republic of Korea

**Keywords:** Design, synthesis and processing, Nanowires

## Abstract

Tubular architecture has been extensively exploited in diverse applications such as solar cells and sensors. However, the synthesis of microtubes with high aspect ratio using polymer templates has been rarely reported. In this study, we designed a facile avenue for the synthesis of well-aligned Au nanoparticle-agglomerate microtubes with an aspect ratio of ~ 30 using a hollow polyetherimide (PEI) template. The combination of wet phase inversion and use of a Cu grid mask enabled straightforward production of a hollow PEI template with vertically aligned tubular architecture. During wet-phase inversion, exchange between a solvent (*N*-methyl-2-pyrrolidone) and a non-solvent (water) occurred at the corners of the square mask cells rather than along their side, thereby producing pores at the corners due to geometrical and entropic factors. The hollow microtubes were comprised of agglomerated Au nanoparticles that coated the inner surfaces of the pores during an electroless plating process performed after wet-phase inversion. This finding is applicable to diverse applications such as sensors and catalysis.

## Introduction

A recent interest in hollow microtubular architecture has surfaced over the past two decades due to the unique physical and chemical properties, such as unprecedented aspect ratio, high specific surface area, and excellent electrical conductivity^1–6^. The tubular microstructures have been exploited in a myriad of applications such as optoelectronics, actuators, sensing, 3D cell microreactors, and catalysis^7–18^. Various approaches have been introduced to fabricate metallic and inorganic microtubes. For instance, ZnO microtubes were manufactured on Si substrates by chemical vapor deposition^19^. Metal precursors were mixed with polyacrylonitrile (PAN) in ethylene glycol and then the electrospun PAN composite was degraded to produce metal microtubes^20^. 2D hexagonal pore arrays were fabricated by the combination of macroporous Si template and deep reactive ion etching, followed by chemical solution method to make ferroelectric microbutes^21^. Rolling-up process achieved the fabrication of diverse metal microtubes^7,16,17,22^.

Among various microtubes, gold (Au) microtubes are particularly useful in a wide range of applications as a possible use in optics, information storage, optoelectronics, separation, sensing, actuators, microelectromechanical devices, and bio sensing devices due to their excellent electrochemical stability, biocompatibility, insusceptibility to oxidation, chemical inertness, and catalysis^1,3–6,23–29^. Au plating can be achieved by electroless plating, electrolytic plating, and e-beam evaporation/lithography^30–34^. Similar to other metal microtubes, gold microtubes have also been fabricated by these various templating/plating methods and special designs. In particular, Au nanoparticles are especially useful to further improve the specific surface area of microsized-structures. For example, Au nanoparticles have been employed as microtube building blocks in biosensors, electronic nanodevices, and applications that employ surface-enhanced Raman scattering, and kapok fiber composite^3,35–37^.

Among various templating methods, the use of a polyetherimide (PEI) template for constructing cylindrical porous structures is promising^38^, and has garnered considerable attention due to its facile fabrication^39–41^. The PEI polymer renders self-growing cylindrical porous structures through a wet phase inversion process^39,41^. However, the random distribution of cylindrical pores in the PEI template is a critical problem. In the present study, we produced a hollow polymer template by a facile process that combined the use of a Cu grid mask with wet phase inversion to achieve a 1D hierarchically aligned Au microtubular structure. This combination led to high aspect ratio over a large area. Owing to its simplicity and versatility, the approach presented herein allows promising strategy for the functionalization of tubular structures.

## Experimental

### Materials

Analytical grade mercaptosuccinic acid powder (TCI, Japan), 1-hexadecanethiol (Sigma-Aldrich Co., St. Louis, MO, USA), tin(II) chloride (SnCl_2_, Mallinckrodt Baker Inc., Phillipsburg, NJ, USA), trifluoroacetic acid (Sigma-Aldrich) gamma-butyrolactone (GBL, Acros Organics Co. Belgium), methanol (BNOChem, South Korea), PEI (Sigma-Aldrich), nitric acid (HNO_3_, Sigma-Aldrich), formaldehyde (HCHO, Sigma-Aldrich), N-methyl-2-pyrrolidone (NMP, Sigma-Aldrich), silver nitrate (AgNO_3_, Sigma-Aldrich), sodium sulfite (Na_2_SO_3_, Sigma-Aldrich), and sodium bicarbonate (NaHCO_3_, Junsei Chemical Co. Japan) were used as received. A 2000-mesh square Cu grid was purchased from Gilder Grids (UK) and used as a mask. The walls of the grid were 5 µm thick, and the openings were 7.5 µm × 7.5 µm in size. Oromerse gold electroless plating solution was purchased from Technic Inc. (Cranston, RI, USA).

### Preparation of masking with thiol treatment

For treatment of masking, 12 mg of mercaptosuccinic acid and 20 mg of 1-hexadecanethiol solution were dissolved in 40 mL of ethanol for hydrophilic and hydrophobic treatment of the Cu grid mask, respectively (Fig. S1). Subsequently, the Cu grid mask was immersed into the solution for 2 h. After treatment, the treated mask was carefully retrieved.

### Fabrication of aligned PEI template

A 10 wt% solution of PEI for fabrication of finger-like microstructures was prepared by dissolving PEI in a solvent mixture (NMP:GBL = 6:4 vol%). The mixture containing NMP and viscous GBL was used as a solvent for wet phase inversion, with GBL controlling the viscosity. The solution was stirred at 70 °C for 5 h. The solution was then cast on a glass slide substrate by doctor-blading. Tape was utilized to control the depth of the solution. After casting at room temperature, a PEI membrane was obtained, onto which the thiol-treated mask was immediately placed. Then, it was immersed in a coagulation medium with water. The solution was inversed from the liquid phase to the solid phase over the course of 4 h. Thereafter, the membrane was immersed in methanol for 1 day, followed by thermal treatment at 230 °C for 15 h to attain a smooth membrane surface.

### Preparation of Au structure in template by electroless plating

Electroless plating involves oxidation and reduction (Fig. S2). In the first step of this process, the membrane was wetted with methanol and then immersed in a 1:1 (v/v) methanol–water solution containing 0.025 M SnCl_2_ and 0.07 M trifluoroacetic acid for 45 min. The membrane was rinsed with methanol. After sensitization with Sn^2+^, the membrane was activated in ammoniacal AgNO_3_ solution (0.029 M) for 5 min. The membrane was then washed with methanol. For Au plating, the membrane was immersed in an Au plating bath (pH 10) consisting of 7.9 × 10^−5^ M Na_3_Au(SO_3_)_2_, 0.127 M Na_2_SO_3_, and 0.625 M formaldehyde at 5 °C. The membrane was cleaned in dilute HNO_3_ solution for 12 h and immersed in methanol. Then, the membrane was immersed in NMP for 1 day to remove the PEI template, and the remaining array was rinsed with deionized-water.

### Characterization

Scanning electron microscopy (SEM, APREO, FEI Co., Hillsboro, OR, USA) with an energy dispersive spectroscopy (EDS) at the Center for Advanced Materials Analysis was performed to investigate the morphologies of the template and gold arrays. The samples on the carbon surfaces were sputter-coated with Pt/Pd. For cross-sectional SEM images, the polymer template was cryo-fractured in liquid nitrogen whereas the aligned Au microtubes were easily detached with tweezers.

Confocal microscopy (FluoView FV1000, Olympus Corp., Japan) was carried out for two (x, y)-dimensional high-resolution imaging of histocultured whiskers with laser excitation at 470 nm to observe the morphology of the Cu mask.

## Results and discussion

**Figure 1:**
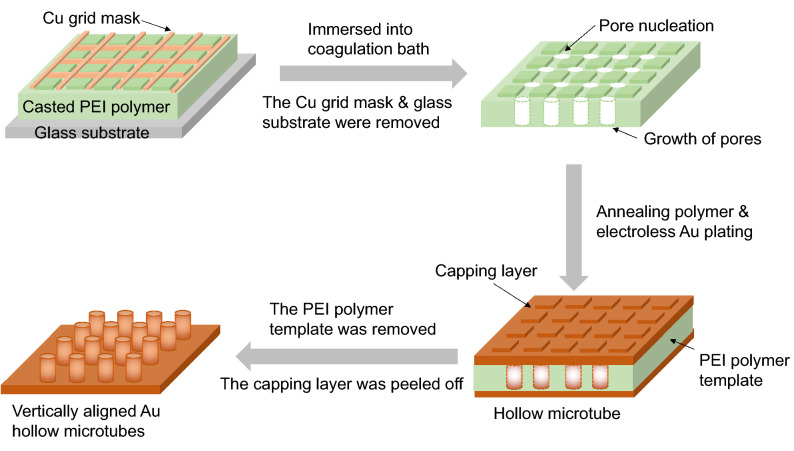
Vertically aligned gold microtube fabrication process.

**Figure 2:**
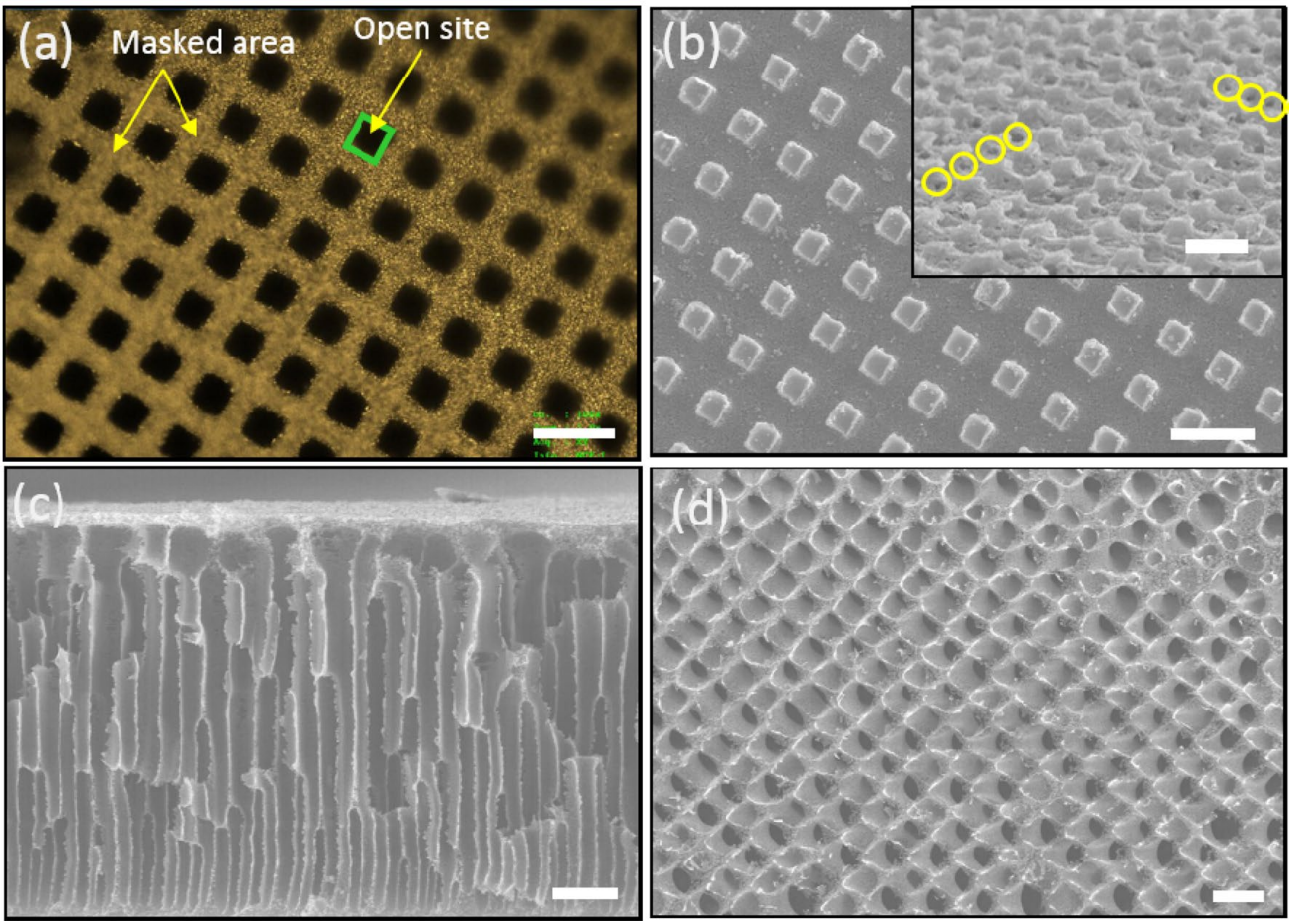
Confocal images of Cu mask (**a**) and SEM images of PEI polymer template (**b**–**d**): (**b**) top view of PEI template (inset: tilt image of PEI template, with yellow circles indicating the nucleation sites), (**c**) cross section of PEI template, and (**d**) highly aligned interior of PEI template after 5 min of oxygen plasma etching. The scale bar is 15 μm.

Highly ordered Au arrays were fabricated by pairing the PEI templating with electroless Au plating, as detailed in Fig. 1. A continuous and uniform porous PEI membrane film as a sacrificial template with an ordered 1D structure was prepared via phase inversion. This phase inversion occurred via the exchange process between a solvent (NMP) and a nonsolvent (DI-water) in a coagulation bath. Phase inversion resulted from transformation of an initially thermodynamically stable polymer solution to an unstable state via the solvent/nonsolvent exchange during coagulation. The resulting membrane structure was composed of a uniform cylindrical pore (honeycomb) structure with a pore diameter range of 3–5 μm. However, the cylindrical porous polymer film fabricated by wet phase inversion generally exhibit a random distribution of pores^42^. In this work, homogeneously aligned structures were obtained by placing a copper mask (Fig. 1a) onto the PEI solution prior to immersion; this process induced tunable and aligned nucleation sites for pore generation on the PEI template film. After the wet phase inversion of the copper mask/PEI solution sample, the mask was easily detached with tweezers. Then, a thin coat of Au was deposited onto the remaining porous template by electroless Au plating. The sacrificial polymer template was removed by NMP. Through this process, well-aligned hollow Au rods were successfully manufactured as shown in Fig. 2.

Visual observation of polymer templates has been routinely carried out through SEM and confocal microscopy. Figure 2b shows a top view of the well-ordered patterns on the PEI film morphology after detachment of the copper mask in the coagulation bath. The regular polymer prominence and depression morphology by mask were observed on the top of PEI polymer film. The tilt inset of Fig. 2b shows the prominent/depressed structure with a nucleation pore marked by yellow circles. The open site (prominent site) underwent the phase inversion between the solvent and the non-solvent. The morphology was confirmed with 2D images obtained by confocal microscopy (Fig. S3). A well-aligned porous structure in the polymer template with copper masking (Fig. S3a–c) and an irregular pore structure without copper masking (Fig. S3d–f) via wet phase inversion were observed. The regularly spaced pore nuclei were observed over a large area on the top of polymer film. The pore nucleation sites were thought to be located at the edge (corner) of the square mask cells due to a geometric effect. The probability of wet phase inversion at the edges of each mask cell was higher than that along the sides of the mask cells.

Figure 2c shows a cross section of the cylindrical PEI templates with no pore distortion owing to the Cu mask. Figure S4 shows the free-standing structure of the Au micro-rods without distortion or damages following dissolution of the polymer template by solvent or electroless Au plating. The large specific surface area could facilitate the application of diverse catalysts and electrochemical devices. To further probe regularity of the aligned cylindrical structure from top to bottom, oxygen plasma etching was performed. Figure 2d shows the interior morphology of the cylindrical PEI template after 300 s of oxygen plasma etching over the top of the template. This indicates a perfectly aligned interior pore structure. Figure S5 exhibits the SEM images of the interior structure of the polymer template as a function of etching time prior to Au electroless plating. Some top prominent structures remained after 30 s of etching (Fig. S5a). At 60 s of etching time of the oxygen plasma, pores were formed in the top layer of polymer template between the prominences (Fig. S5b). Finally, the well-aligned porous arrays were obtained at 120 s of etching time (Fig. S5d). Figures 2a–d demonstrate that the sacrificial template allowed formation of the well-aligned interior structure.

**Figure 3:**
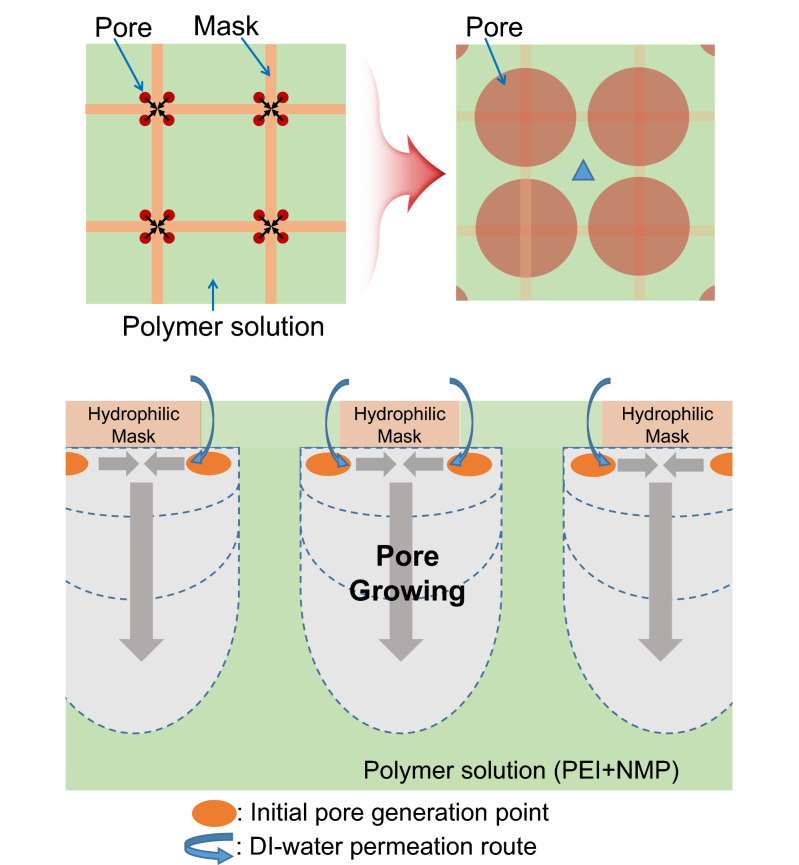
Wet phase inversion process: Initial pore generation at edges of masks and pore growth underneath intersection of mask lines.

The porous structure was generated underneath intersection of mask lines via initial pore generation at edges of masks as shown in Fig. 3. The initially produced small pores at edges were coalesced nearest to each pore, thereby leading to large porous structure underneath the intersection of mask lines. However, it might have been located at the center of polymer solution (blue triangle) in Fig. 3, if the mask lines were substantially thick due to closest distance between initial pores.

**Figure 4:**
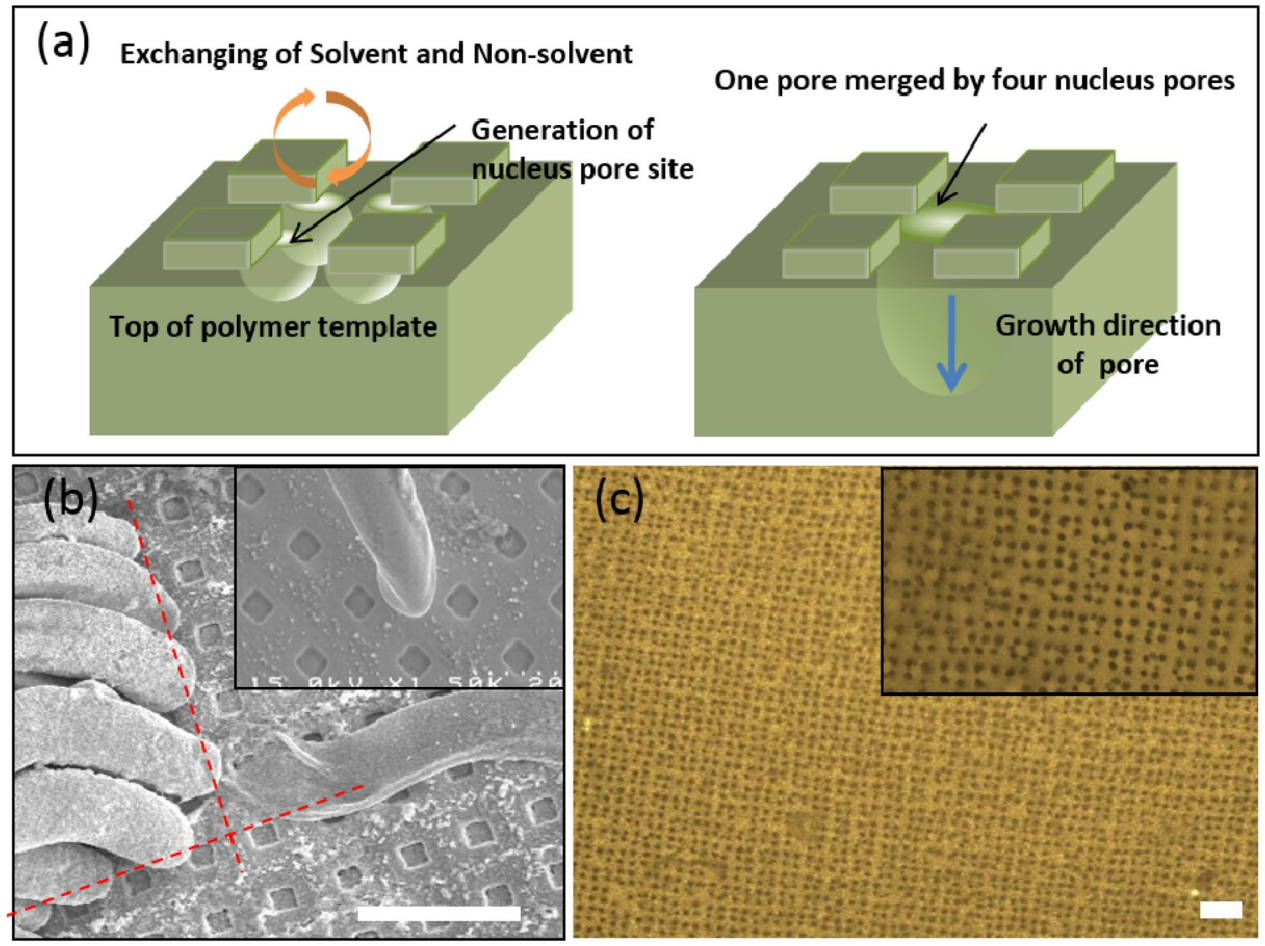
Schematic of mechanism and SEM images (scale bar is 20 μm): (**a**) pore generation mechanism, (**b**) a bundle of Au-capped rods (inset: single Au rod), (**c**): confocal image of nucleation pores on PEI membrane (inset: magnified images).

The mechanism of pore nucleus generation on the PEI template via phase inversion is illustrated in Figs. 3 and 4a. Prior to phase inversion, the pore nuclei emerged at the edges (corners) of the square cells of the mask grid on the upper surface of the PEI polymer film (Fig. 4). This phenomenon facilitated inversion at the corners of the square cells, but not near their center. Upon addition of the solvent, the polymer-phase with a solvent underwent a transition from a homogeneous mixture to a polymer-rich and polymer-poor phases. Pores formed in the polymer-poor phase and formed preferentially at the cell corners because the geometric and entropic conditions there were more favorable for pore formation than they were along the sides of the cells. The pore nuclei generated at the corners of four mask cells merged to form a larger pore (3 μm in diameter), which spanned the full vertical length of the PEI film. Au rods were generated via electroless plating of the inner pore walls, which corresponded to the open areas of the Cu mask grid.

**Figure 5:**
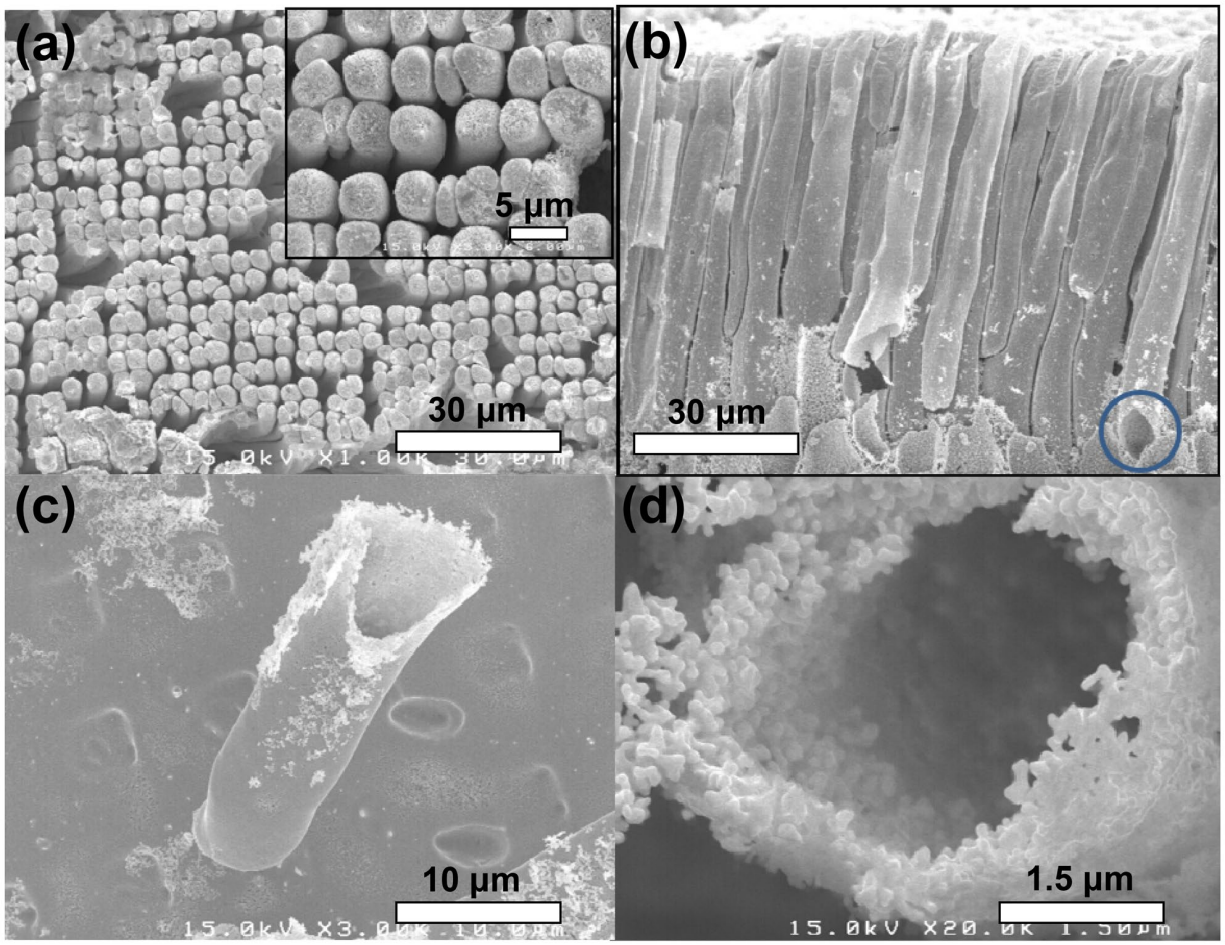
SEM images of gold microtubes. Top view (**a**) and cross sectional view (**b**) of aligned Au microtube. Single Au microtube (**c**,**d**).

The PEI template was annealed at 230 °C for 15 h to remove some porous sites of the PEI wall, thereby achieving a smooth wall of interior template for the hollow Au rod^43^. The prominence and depression structures were obtained by Au plating, as shown in Fig. S4. The SEM images indicate that the polymer template remained undamaged without distortion during annealing and electroless Au plating. As shown in Fig. 5b, a thin Au capping layer grew on the rod during electroless Au plating. After dissolving the polymer template in the solvent (NMP), well-aligned hollow Au rod arrays with a diameter of 3–5 μm and height of 80–90 μm (Fig. 5b) were obtained by carefully peeling the capping layer on the top of Au microtubes (Fig. S6). The Au capping layer was easily removed with tweezers due to the empty space (originally the polymer template) between the Au capping layer and Au rod arrays. The aspect ratio of 20–30 was analogous to the dimensions of the template. The rods were rarely collapsed by a capillary force. Gold nanoparticles (submicron) were highly packed and assembled into a hollow tubular morphology with a micrometer scale of 3–5 μm in Figs. 5c,d. Small metal nanoparticles tend to aggregate to minimize their surface area. In this study, the rod structures were chiefly composed of Au (Fig. S7), following the removal of the template.

## Conclusion

The facile fabrication of well-aligned hollow Au rods was achieved using a PEI polymer template and electroless Au deposition. A cylindrical porous polymer template was simply generated via phase inversion. This procedure is readily amenable to scaling up and will allow for the exploration of electronic, optical, chemical and physical properties for practical applications. The results of this work may serve as a reference for nanotube fabrication via lithography using a nano-patterned Cu mask. These nanotubes are useful in nanoscale applications, such as nanoelectronics, sensors, and catalysts.

## Supplementary information

Supplementary file1
