# Supplementary material for: Vertically aligned nanostructured gold microtube assisted by polymer template with combination of wet phase inversion and Cu grid mask
Source: Sci Rep. 2020 Oct 2;10:16420. doi: 10.1038/s41598-020-73506-1 (PMC7532224; doi:10.1038/s41598-020-73506-1)
Supplement: Supplementary file 1 — Supplementary file1 [file 41598_2020_73506_MOESM1_ESM.docx]

Vertically Aligned Nanostructured Gold Microtube Assisted by Polymer Template with Combination of Wet Phase Inversion and Cu Grid Mask

Soohyun Kim^1^, Keon-Soo Jang^2^*

^1^ *LG Chem. Magokjungang 10-ro, Gangseo-gu, Seoul, 07796, Republic of Korea*

^2^ *Department of Polymer Engineering, School of Chemical and Materials Engineering, The University of Suwon, Hwaseong, Gyeonggi-do 18323, Republic of Korea*

**CORRESPONDING AUTHOR FOOTNOTE** *To whom correspondence should be addressed. E-mail: ksjang@suwon.ac.kr

**Supplementary information**


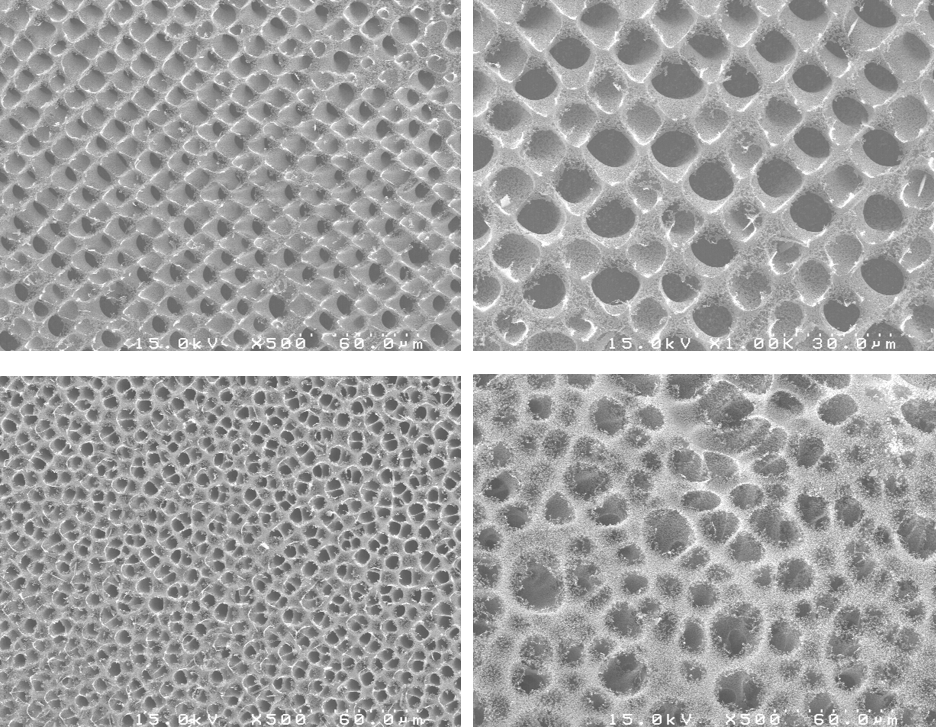


**(a)**

**(b)**

**(c)**

**(d)**

Figure S1. SEM images of porous structure with mercaptosuccinic acid-treated (a,b) and hexadecanethiol-treated (c,d) copper masking after oxygen plasma etching: 0 sec (a,c) and 120 sec (b,d)


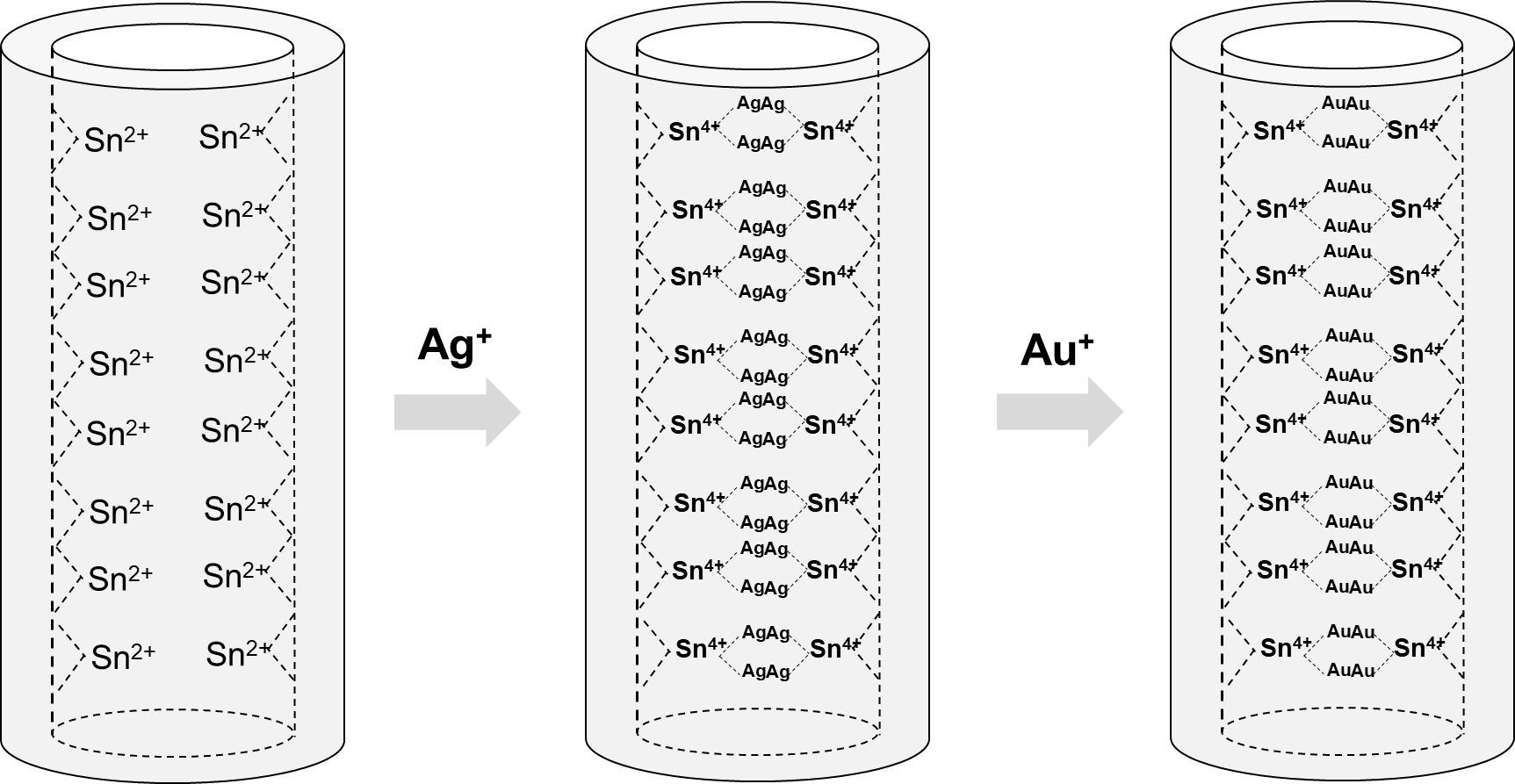


Figure S2. Scheme of electroless Au plating

The membrane was immersed in SnCl_2_ and trifluoroacetic acid with the methanol–water mixture solution. The surface of polymer membrane was functionalized by Sn^2+^. The membrane was then activated by the AgNO_3_ solution. The surface was transitioned from Sn^2+^ to Ag. The membrane was immersed in the Au plating bath with Na_3_Au(SO_3_)_2_ and Na_2_SO_3_, and in formaldehyde, thereby producing Au.

**(a)**

**(b)**

**(c)**

**(d)**

**(f)**

**(d)**


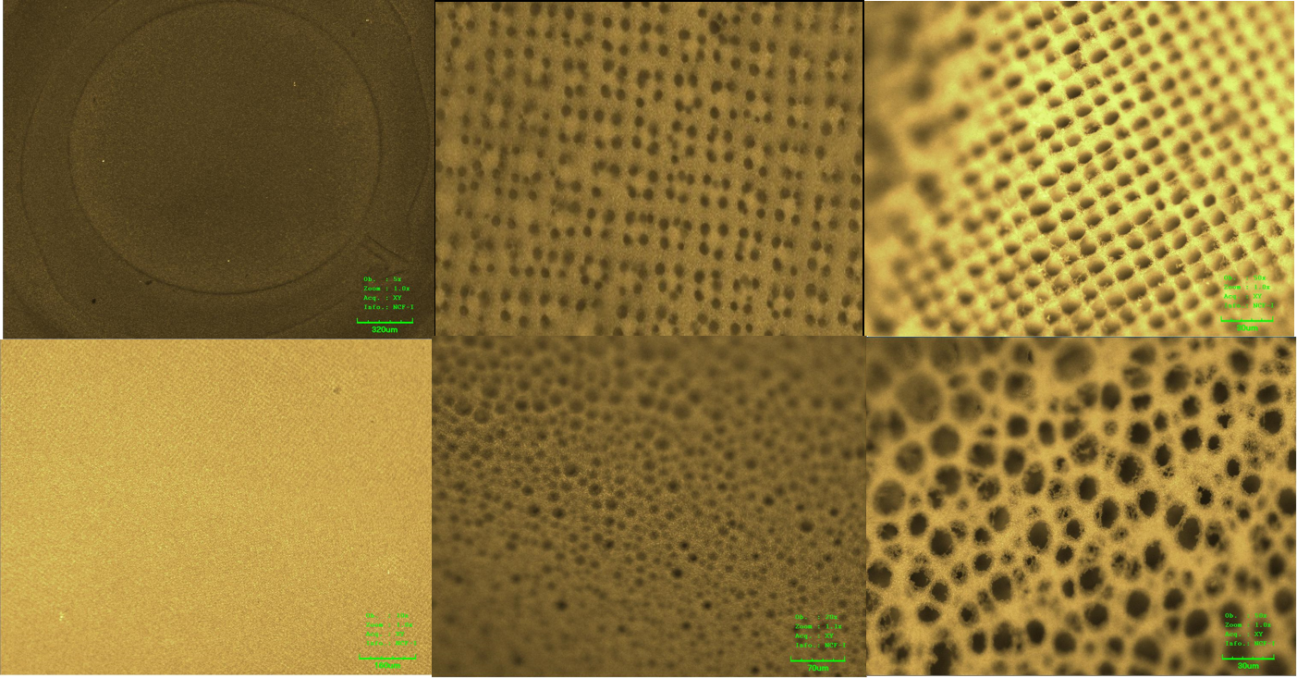


Figure S3. Confocal microscopy images of polymer templates with (a,b,c) and without copper masking (d,e,f) as a function of oxygen plasma etching: 0 sec (a,d), 60 sec (b,e), and 120 sec (c,f)


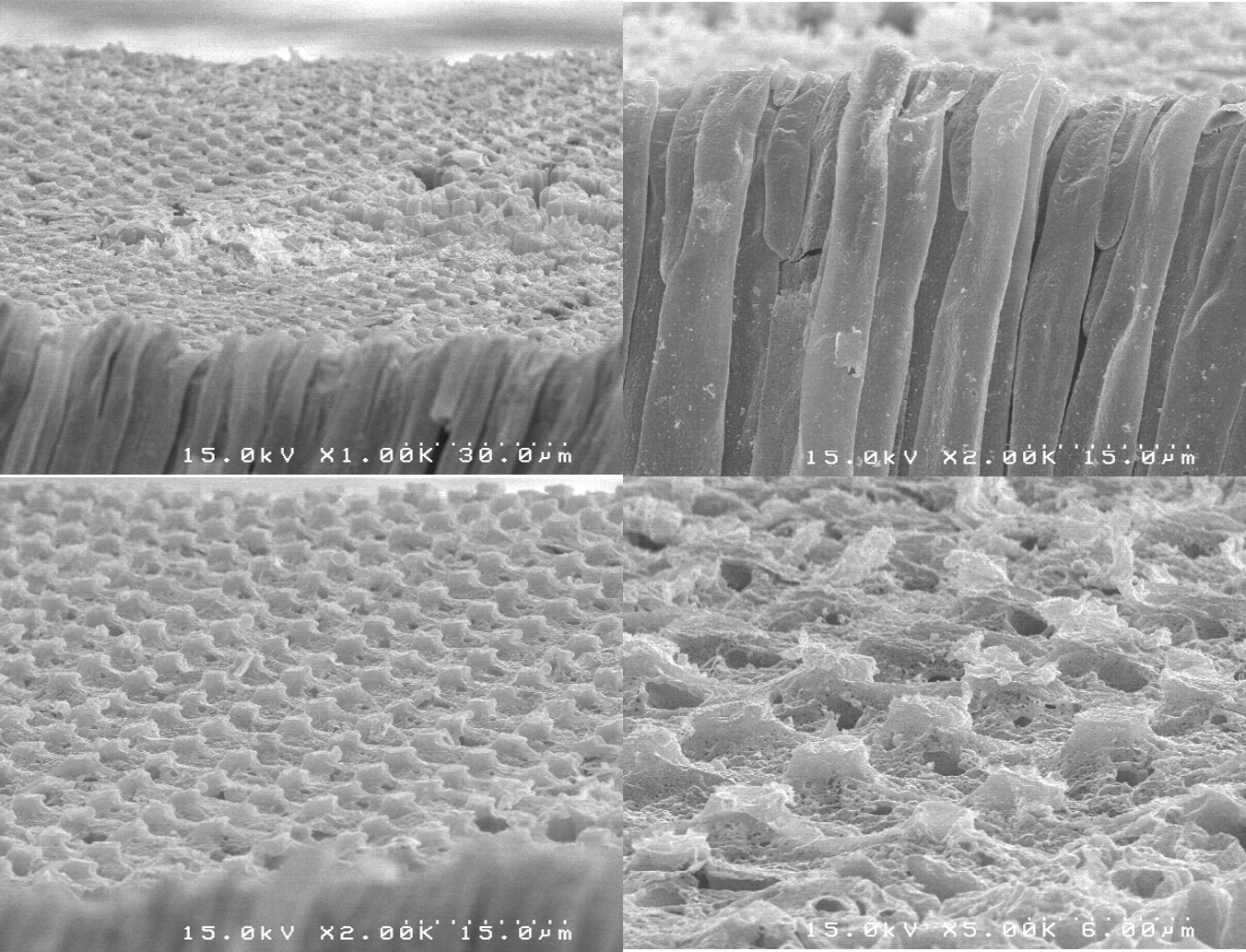


**(a)**

**(b)**

**(c)**

**(d)**

Figure S4. Side view SEM images of generated Au electroless plating after dissolving the polymer template


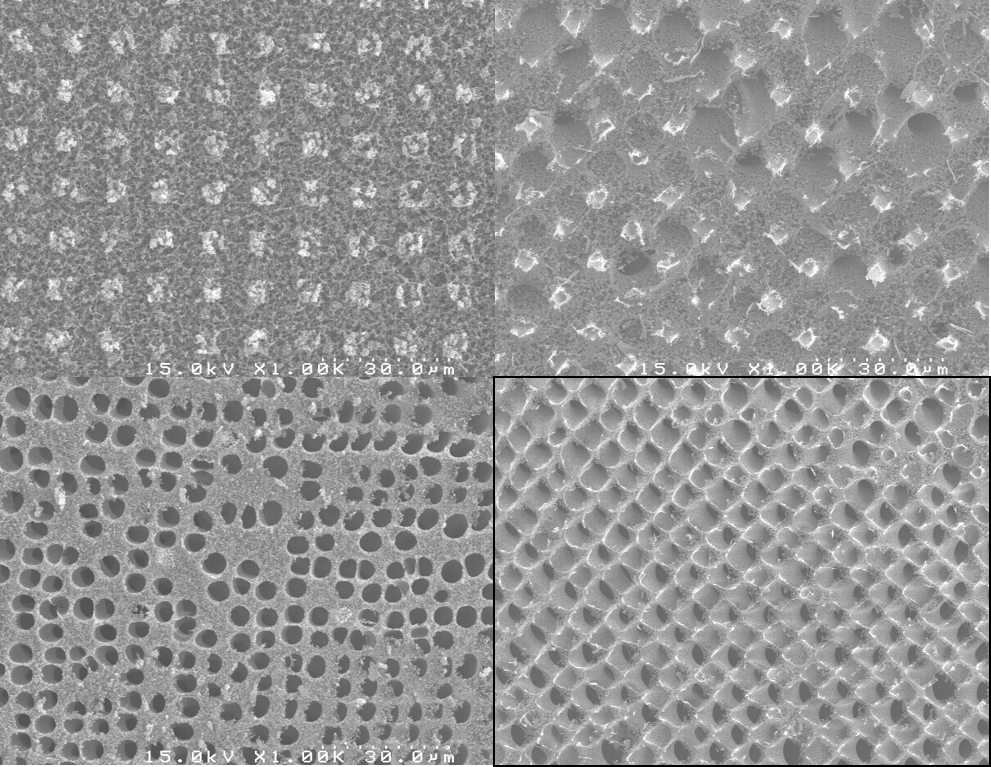


**(a)**

**(b)**

**(c)**

**(d)**

Figure S5. SEM images of PEI template before Au electroless plating. Plasma oxygen etching time: (a) 30 sec, (b) 60 sec, (c) 90 sec, and (d) 120 sec.


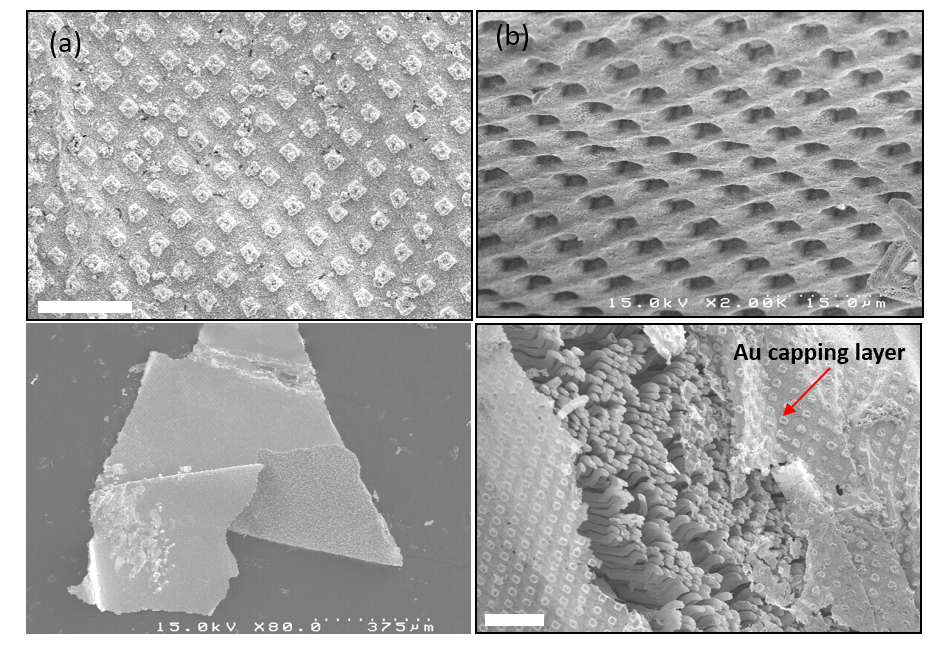


**(a)**

**(b)**

**(c)**

**(d)**

Figure S6. SEM images of Au microtubes with Au capping layer after removing the polymer template: (a) top view, (b) bottom view, (c) removed capping Au layer by tweezers, and (d) Au microtubes beneath Au capping layer.


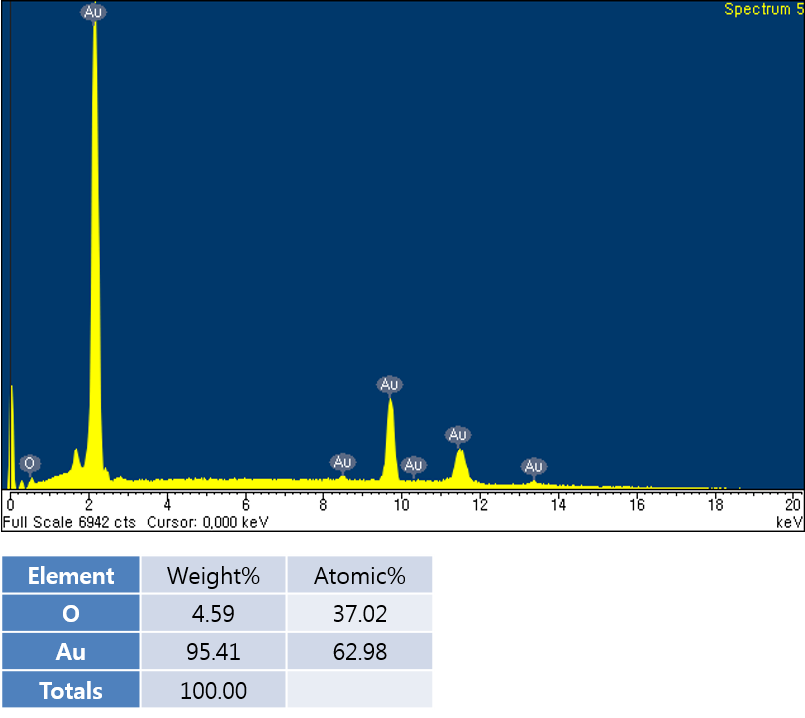


Figure S7. EDS spectra of Au microtubes after removing the polymer template by solvent
